# A yeast-based system to study SARS-CoV-2 M^pro^ structure and to identify nirmatrelvir resistant mutations

**DOI:** 10.1371/journal.ppat.1011592

**Published:** 2023-08-31

**Authors:** Jin Ou, Eric M. Lewandowski, Yanmei Hu, Austin A. Lipinski, Ali Aljasser, Mariliz Colon-Ascanio, Ryan T. Morgan, Lian M. C. Jacobs, Xiujun Zhang, Melissa J. Bikowitz, Paul R. Langlais, Haozhou Tan, Jun Wang, Yu Chen, John S. Choy

**Affiliations:** 1 Department of Biology, School of Arts and Sciences, The Catholic University of America, Washington, Washington DC, United States of America; 2 Department of Molecular Medicine, Morsani College of Medicine, University of South Florida, Tampa, Florida, United States of America; 3 Department of Medicinal Chemistry, Ernest Mario School of Pharmacy, Rutgers, the State University of New Jersey, Piscataway, New Jersey, United States of America; 4 Department of Medicine, College of Medicine, University of Arizona, Tucson, Arizona, United States of America; 5 Drug Discovery Department, Moffit Cancer Center, Tampa, Florida, United States of America; Texas A&M University, UNITED STATES

## Abstract

The SARS-CoV-2 main protease (M^pro^) is a major therapeutic target. The M^pro^ inhibitor, nirmatrelvir, is the antiviral component of Paxlovid, an orally available treatment for COVID-19. As M^pro^ inhibitor use increases, drug resistant mutations will likely emerge. We have established a non-pathogenic system, in which yeast growth serves as an approximation for M^pro^ activity, enabling rapid identification of mutants with altered enzymatic activity and drug sensitivity. The E166 residue is known to be a potential hot spot for drug resistance and yeast assays identified substitutions which conferred strong nirmatrelvir resistance and others that compromised activity. On the other hand, N142A and the P132H mutation, carried by the Omicron variant, caused little to no change in drug response and activity. Standard enzymatic assays confirmed the yeast results. In turn, we solved the structures of M^pro^ E166R, and M^pro^ E166N, providing insights into how arginine may drive drug resistance while asparagine leads to reduced activity. The work presented here will help characterize novel resistant variants of M^pro^ that may arise as M^pro^ antivirals become more widely used.

## Author summary

The SARS-CoV-2 virus has proven to be highly adept at evading the newly developed vaccines. Antiviral drugs provide an important alternative that can reduce disease severity. Nirmatrelvir, an orally available drug approved by the FDA to treat COVID-19, inhibits the SARS-CoV-2 main or 3C-like protease (M^pro^ or 3CL^pro^) and reduces the severity of COVID-19 infections. However, continued use of nirmatrelvir will likely drive the emergence of drug resistant M^pro^ mutations. Our study reports a rapid, inexpensive, and non-pathogenic system using baker’s yeast that can determine if potential M^pro^ mutations may confer drug resistance. Furthermore, we solve the structures of two M^pro^ mutants first characterized in yeast that provide insights into how E166 can change to drive resistance versus leading to inactivity. Our results can aid in advancing our understanding of resistance mechanisms that will be important as nirmatrelvir and other protease inhibitors become more widely used.

## Introduction

The evolution of new SARS-CoV-2 variants that evade vaccines, cause breakthrough COVID-19 infections in vaccinated individuals, and the limited vaccine availability in many parts of the world, highlight the need for complementary approaches [1]. Antiviral drugs provide an important alternative and can contribute to minimizing disease severity and death. The SARS-CoV-2 main or 3C-like protease (M^pro^ or 3CL^pro^) is essential for viral replication and is a promising drug target [2,3]. There have been intense efforts to repurpose or to develop new drugs that directly target M^pro^ [4,5]. In December 2021, emergency authorization use of Paxlovid to treat COVID-19 was granted by the US Food and Drug Administration [6]. Paxlovid is a combination of the M^pro^ inhibitor, nirmatrelvir, and the cytochrome CYP3A inhibitor, ritonavir, which slows metabolism of nirmatrelvir [7,8]. There are several other M^pro^ inhibitors in clinical trials, including PF-07304814, the phosphate form of PF-00835231[9,10], and ensitrelvir [11]. As M^pro^ inhibitors become more widely used the emergence of resistant mutations will increase as greater selection pressure is present in the population.

Knowledge of resistant mutants can inform on drug design modifications to identify new drugs that target resistant variants. However, standard approaches to characterize resistant mutants using live virus [12], recombinant proteins, and in vitro assays can be highly limiting due to infrastructure requirements, cost, and time [13]. Here we report a yeast system that is non-pathogenic, rapid, inexpensive, and reports on M^pro^ activity and drug resistance simply by measuring yeast growth. Using this assay, we found that compared to wild-type, the E166R mutation conferred strong nirmatrelvir resistance (*K*_*i*_ > 1000-fold). As the E166 site appears to be a hot spot for drug resistance from *in vitro* viral evolution experiments [14,15], we solved the structures of two substitution mutants M^pro^ E166N and M^pro^ E166R, revealing how E166 mutations may compromise activity versus drug resistance, respectively. Furthermore, we tested two known resistant mutants identified by *in vitro* SARS-CoV-2 evolution experiments [14,16,17] and find that they are resistant in the yeast assay. Our results demonstrate the yeast system can be a reliable tool to determine the activity and drug responses of M^pro^ mutants. Results from the yeast assays can help rapidly prioritize mutants for further analysis using more resource intensive systems. In doing, so we can efficiently test M^pro^ mutants as they arise in the population and aid in mitigating COVID-19 infections.

## Results

### SARS-CoV-2 M^pro^, PL^pro^, spike, and helicase proteins are toxic in *S*. *cerevisiae*

**Fig 1:**
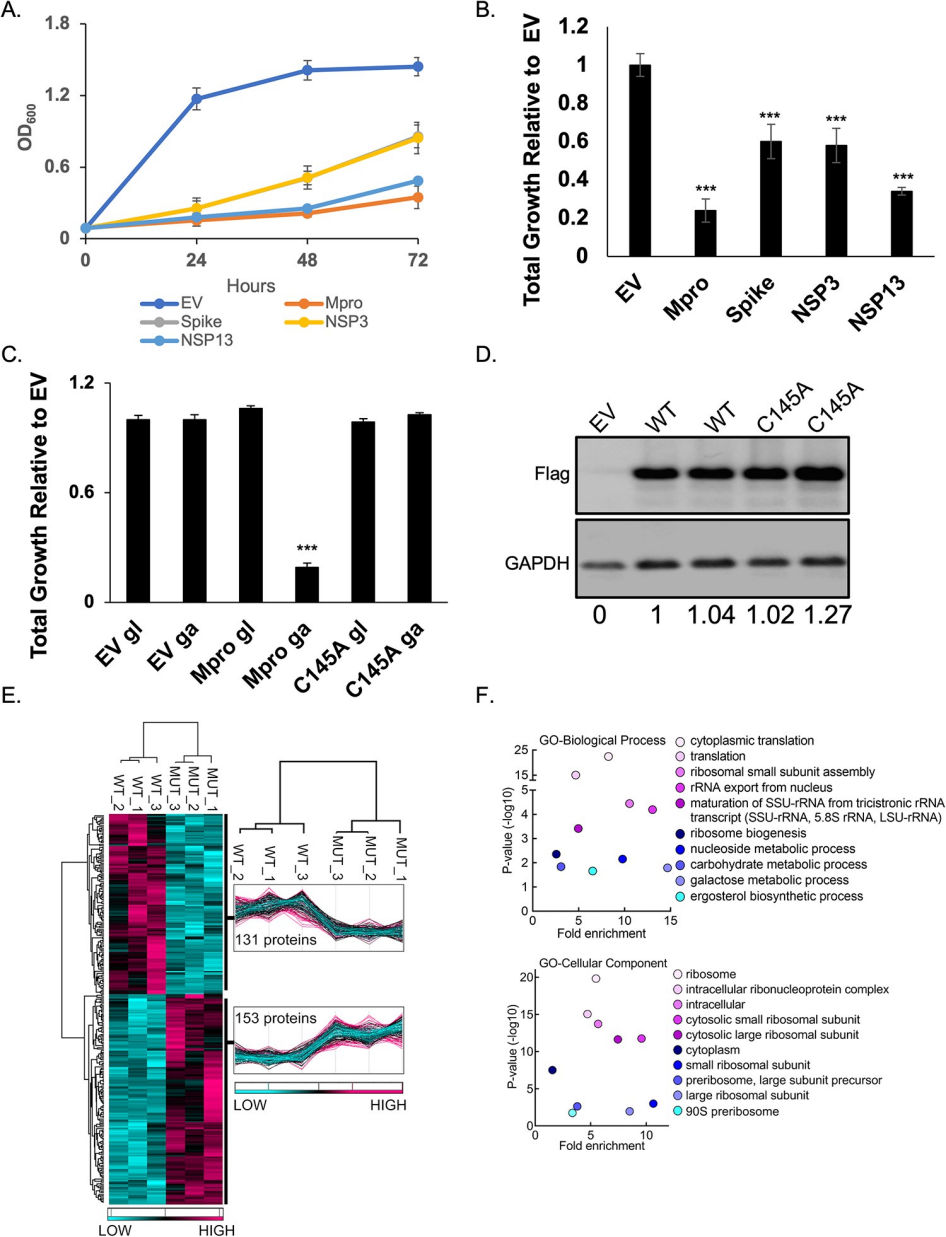
M^pro^ confers a significant reduction in growth in yeast caused by decreases in a variety of cellular proteins. A) The indicated SARS-CoV-2 genes regulated by a galactose inducible promoter were expressed in yeast and conferred growth defects compared to empty vector (EV). B) Bar graph shows the total growth of cultures after 72 hours normalized to EV. C) Galactose-induced (ga) expression of the catalytically inactive M^pro^ C145A mutant (C145A ga) does not confer a growth reduction compared to WT (M^pro^ ga). When grown in glucose (gl) all three strains grew equally well. D) Protein levels of the M^pro^ C145A mutant and wild-type M^pro^ (WT) are comparable. Shown are two biological replicates for each form of M^pro^. Ratios of Flag:GAPDH signals relative to M^pro^ WT is shown at the bottom of each lane. E) Total protein lysates made from yeast expressing the wild-type M^pro^ (WT) or M^pro^ C145A mutant (MUT) were subjected to mass spectrometric analyses revealing 153 proteins were higher in abundance in the mutant relative to the wild-type. F) Gene Ontology (GO) analyses indicates an enrichment of proteins with functions in translation that are significantly reduced in the presence of M^pro^ versus M^pro^ C145A. Plots in A, B, C show averages from three biological replicates and error bars are standard deviations. (***) indicates differences (p<0.001)between EV and tested genes.

Six SARS-CoV-2 (Wuhan-Hu-1) NSPs and the structural genes were expressed from a high copy plasmid [18] to determine if any would result in growth effects (Figs 1A and S1A). We observed no marked growth phenotypes as determined by spot tests when M, E, N, NSP7, NSP8, or NSP12 were expressed (S1B Fig). In contrast, spot tests revealed nearly a complete absence of growth when cells expressed NSP3 (PL^pro^), NSP5 (M^pro^ or 3CL^pro^), NSP13 (Helicase), and spike (S1B Fig). Analysis of growth profiles of cells expressing PL^pro^, M^pro^, Helicase, and spike showed all four genes caused a reduction in growth. Previous reports have shown that expression of the SARS-CoV-1 PLpro reduces yeast growth [19]. M^pro^ and the Helicase were the most toxic conferring a ~70 to 80% reduction in total growth by 72 hours compared to cells carrying empty vector (Fig 1A and 1B). As M^pro^ is highly conserved between classes of coronavirus and a key drug target we focused our efforts on using the yeast system to study M^pro^ structure and function.

### Growth defect conferred by M^pro^ expression depends on its catalytic activity and associated with decreased abundance in essential and non-essential yeast proteins

To determine if the growth reduction depended on M^pro^ proteolytic activity we constructed a catalytic mutant of M^pro^ by replacing the key cysteine at position 145 to an alanine, which prevents the initial protonation step needed for peptide bond hydrolysis [20,21]. Liquid growth assays showed that yeast expressing the M^pro^ C145A mutant grew as well as the yeast control carrying empty vector (Fig 1C). In contrast, all three strains grew similarly in glucose, which represses expression of M^pro^ (Fig 1C). Western analysis showed that yeast expressed similar levels of wild-type and M^pro^ C145A mutant (Fig 1D). These results demonstrate that the growth reduction observed in yeast expressing M^pro^ is dependent on its proteolytic activity.

Next, we measured the relative abundance of proteins in yeast expressing M^pro^ compared to yeast expressing the M^pro^ C145A catalytic mutant to determine the mechanism(s) that lead to loss of cell viability. Whole cell lysates were made from three independent cultures of cells expressing wild-type M^pro^ or the catalytic M^pro^ C145A mutant (Figs 1E and S2). The biological replicates were highly reproducible, and we observed peptides from 153 proteins (S1 Table) were significantly reduced in yeast expressing M^pro^ compared to the M^pro^ C145A mutant (Fig 1E.) Gene ontology analysis revealed an enrichment for genes with functions in translation (Fig 1F). In particular, multiple ribosomal proteins and translational regulators were reduced. There were a number of proteins that were significantly enriched in the M^pro^ catalytic mutant with functions in a variety of activities beyond translation (S1 Table) and several are known to be essential (S1 Table). Furthermore, approximately 16% (25/153) of reduced proteins contained at least one potential canonical M^pro^ site (LQ/S, G, A) (S2 Table) suggesting that some may be direct substrates of M^pro^ [22,23]. Another 36% (55/153) of reduced proteins carried non-canonical sites in which LQ was followed by any amino acid besides S, G, or A. These results show that expression of M^pro^ leads to decreases in a variety of proteins and eventual loss of translation that is likely the cause of the growth defects.

### Nirmatrelvir restores growth to yeast expressing M^pro^ from multiple coronaviruses

**Fig 2:**
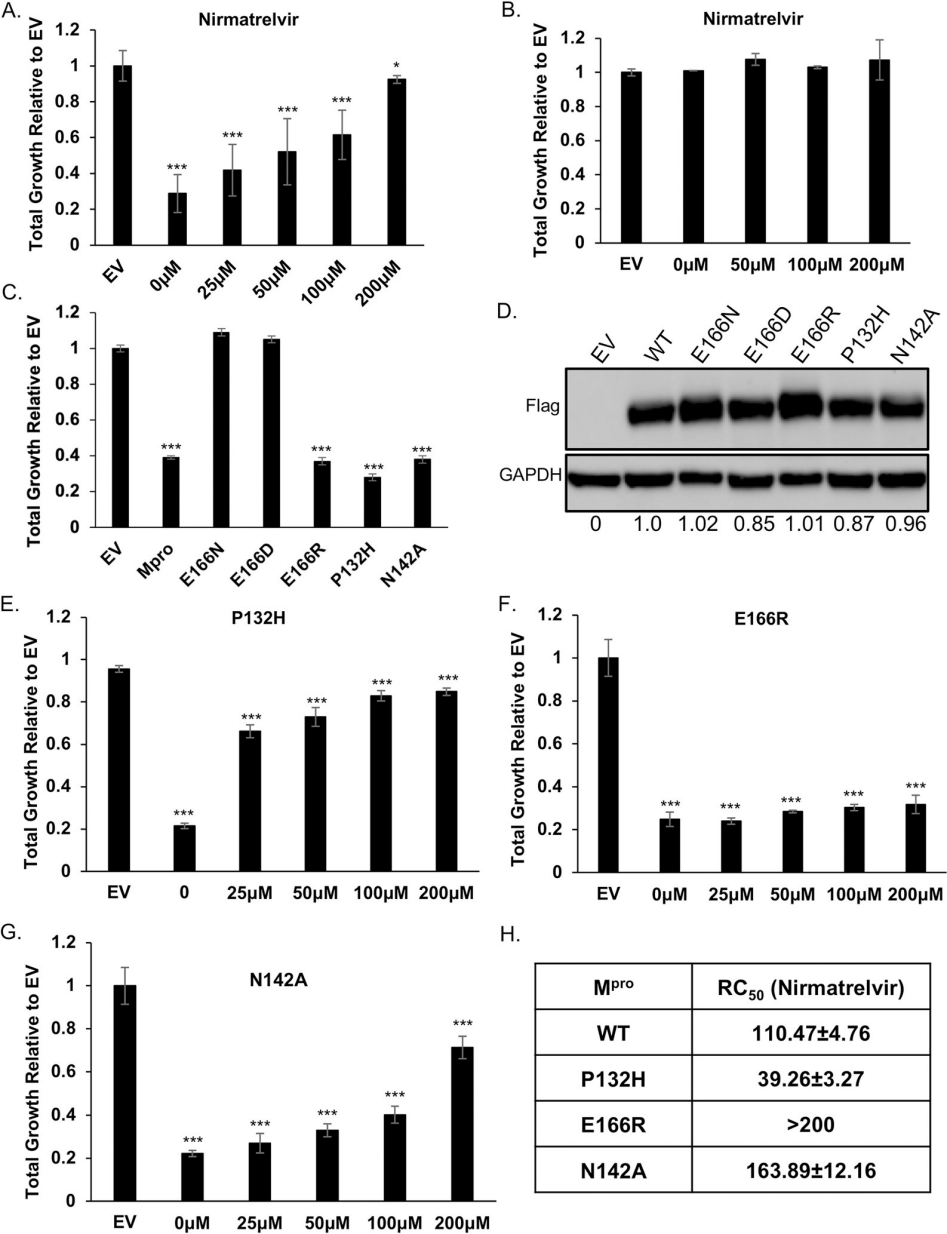
Yeast growth assays identify nirmatrelvir resistant M^pro^ mutants. A) Total growth of cultures after 72 hours expressing M^pro^ in the presence of increasing doses of nirmatrelvir normalized to growth of yeast carrying empty vector (EV) are plotted. Growth is restored by nirmatrelvir in a dose dependent manner. B) No growth effects are observed in cells treated up to 200μM nirmatrelvir. C) Yeast expressing substitutions E166D and E166N grow as well as EV but E166R, P132H, and N142A results in significant growth reduction comparable to wild-type M^pro^. D) Western analysis shows that mutants and wild-type M^pro^ are expressed at comparable levels. Ratios of Flag:GAPDH signals relative to M^pro^ WT is shown at the bottom of each lane. E—G) Cells expressing P132H and N142A remain sensitive to nirmatrelvir, indicated by growth recovery, but E166R appears to be resistant as there is a lack of growth even when treated with 200μM of nirmatrelvir. H) RC_50_ measurements of each mutant in response to nirmatrelvir treatment. For all experiments, at least three biological and three technical replicates were performed. Error bars represent standard deviations. (*, p<0.01; ***, p<0.001) indicates differences compared to EV.

Considering that the growth reduction conferred by M^pro^ activity is dependent on its proteolytic activity we tested if treating yeast with nirmatrelvir, would suppress the growth reduction. We tested nirmatrelvir at several concentrations and observed no cytotoxic effects (Fig 2B). Treating cells with increasing doses of nirmatrelvir led to a corresponding increase in growth (Fig 2A). At 100μM and 200μM of nirmatrelvir, growth was restored to similar levels as cells carrying empty vector (Fig 2A). As a qualitative measure to compare the effects of nirmatrelvir, we estimated the concentration of drug required to restore 50% of growth (RC_50_) relative to that of untreated M^pro^ expressing cells. Based on this criterion we calculated RC_50_ for nirmatrelvir to be 110.47 ± 4.76μM (Fig 2A and 2H). To determine if M^pro^ from other coronaviruses could be studied similarly, we tested the recent Omicron variant, M^pro^ P132H, which is currently the dominant form of M^pro^, and M^pro^ from SARS-CoV-1 and Bat-CoV-HKU9. We observed that in all cases M^pro^ conferred a significant growth reduction (Figs 2C and S3). Nirmatrelvir has been reported to have broad M^pro^ specificity [7,9]. Consistent with this work, we observed that nirmatrelvir could restore growth in yeast expressing M^pro^ from all three forms of M^pro^ (Figs 2E, 2H, and S3).

### Characterization of potential nirmatrelvir resistant mutations in M^pro^

We tested if growth of yeast expressing M^pro^ could be used as an approximation for M^pro^ activity. Thus, providing a system to rapidly determine the effects of mutations on M^pro^ activity and drug resistance. A variety of interactions (H-bonds, salt-bridges, van der waals) mediate binding between the catalytic site of M^pro^ and inhibitors [24–26]. While knowledge of the residues in contact with the inhibitor can inform predictions that may compromise inhibitor binding it is not obvious what amino acid substitutions would maintain M^pro^ activity toward substrate while compromising inhibitor interactions. With our yeast system we can easily test the effect of substitution mutations and rapidly determine if the mutations alter catalytic activity and sensitivity to inhibitor(s) by following growth phenotypes. To determine the feasibility of this approach we focused on E166, and N142 as these two residues form direct interactions with inhibitors and substrates [24,27].

We tested substitutions of E166 with three different amino acids that are yet to be dominant in the population. The following mutants predicted to be conserved (E166D), as the negative charge is maintained but with one less carbon in the side-chain; non-conserved (E166N), as asparagine is uncharged and has one less side chain carbon; and another non-conserved (E166R) substitution in which the arginine side chain is longer and positively charged.

We observed that all three substitution mutants were expressed at levels comparable to wild-type M^pro^ (Fig 2C). Expression of M^pro^ E166D and M^pro^ E166N did not cause a reduction in growth and grew as well as empty vector controls (Figs 2C, 2D and S4A). These results indicate that M^pro^ E166D and M^pro^ E166N may have defects in their enzymatic activities. However, the M^pro^ E166R mutant conferred a growth reduction that matched the wild-type M^pro^, suggesting that its catalytic activity was intact (Figs 2C and S4B). While this manuscript was in preparation it was reported from *in vitro* SARS-CoV-2 evolution experiments that E166A and E166V substitutions conferred nirmatrelvir resistance [14,16,17]. Thus, we tested these two mutants in yeast and observed that both mutants were expressed at comparable levels and conferred a growth reduction similar to wild-type M^pro^ (S5A and S5B Fig).

Next, we challenged cells expressing M^pro^ E166R, E166A, and E166V with increasing concentrations of nirmatrelvir (25μM, 50μM, 100μM, or 200μM) and observed no significant improvement in growth remaining nearly identical to the untreated cultures (Figs 2F, S4B, S5C and S5D). Based on these experiments, the RC_50_ for nirmatrelvir is >200μM compared to wild-type M^pro^ (Figs 2H, S5C and S5D). These results suggest that the E166R, E166A, and E166V mutations confer resistance to nirmatrelvir.

We constructed a substitution at position N142, which is known to contribute to inhibitor and substrate binding [7] and is yet to be present in the population. To inform on the specific substitution to make we used a distantly related M^pro^ from the gamma-coronavirus, IBV, which is conserved but displays slight divergence from SARS-CoV-2 M^pro^ [9]. We replaced N142 with alanine (M^pro^ N142A), as alanine is found in the IBV M^pro^ at the homologous site [28]. We observed M^pro^ N142A was expressed at levels comparable to wild-type and conferred a similar reduction in growth (Fig 2C and S4C) showing that it remained active. The RC_50_ for nirmatrelvir increased modestly by ~1.5-fold (Fig 2G and 2H). These results show that the substitution mutants E166A, E166V, and E166R lead to nirmatrelvir resistance while N142A results in little difference from wild-type and E166N and E166D cause a significant loss in activity.

### *In vitro* protease assays confirm that M^pro^ E166R, E166A, E166V are highly resistant to nirmatrelvir

**Fig 3:**
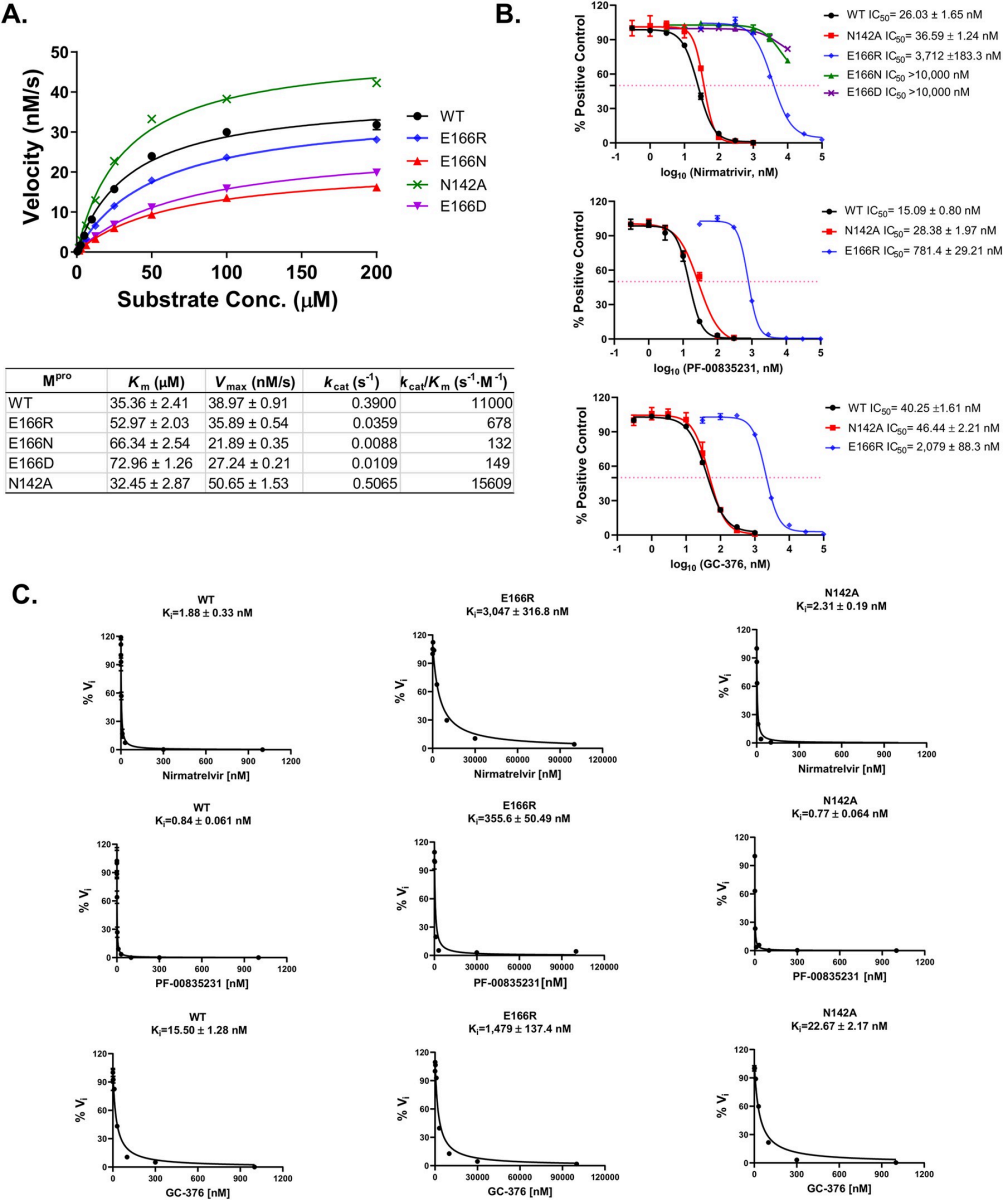
Enzymatic assays demonstrate that E166N and E166D have severe defects in catalytic activity and M^pro^ E166R is highly resistant to nirmatrelvir. A) Michaelis–Menten plot of M^pro^ and its mutants with various concentrations of FRET substrate. The K_m_, V_max_, k_cat_, and *k*_*cat*_*/K*_*m*_ values are shown in the table. B) The IC_50_ plots of nirmatrelvir, PF-00835231. and GC-376 against M^pro^ WT, N142A, E166R, E166N, and E166D. C) K_i_ plots of nirmatrelvir, GC-376, and PF-00835231 against M^pro^, M^pro^ E166R, and M^pro^ N142A.

To determine how well yeast growth assays correlated with standard enzymatic assays we directly measured proteolyitc activity using recombinant M^pro^ WT, E166N, E166D, E166R, E166A, E166V, and N142A. Compared to wild-type M^pro^, the catalytic efficiencies (*k*_*cat*_*/K*_*m*_) of M^pro^ E166R, E166A, and E166V were decreased by ~16-fold, ~8-fold, and ~10-fold, respectively (Figs 3A and S6A). In contrast, E166N and E166D displayed severe reductions in catalytic efficiencies of ~84- and ~74-fold compared to WT, respectively (Fig 3A). On the other hand, M^pro^ N142A displayed a slight increase in catalytic efficiency of ~1.4-fold compared to WT (Fig 3A). To determine the response of the mutants to inhibitors we performed IC50 and Ki measurements. We observed for M^pro^ E166R, E166A, and E166V, increases in IC50’s of ~143-, ~6-, >300-fold for nirmatrelvir compared to WT, respectively (Figs 3B, 3C, S6B and S6C). On the other hand, M^pro^ N142A, only minor increases were observed in IC_50_’s of ~1.4-fold for nirmatrelvir (Fig 3B). The *K*_*i*_ values for the inhibitors in assays with M^pro^ E166R, E166A, and E166V were increased by >1600-, >47-, and >5000-fold for nirmatrelvir, ~423-,~27-, ~790-fold for PF-0085231, and ~37, ~9-, ~38-fold for GC-376, respectively. (Figs 3C and S6C). Nearly no difference in Ki values was observed between wild-type M^pro^ and M^pro^ N142A, ~1.2-fold for nirmatrelvir, ~0.9-fold for PF-0085231, ~1.5-fold for GC-376) (Fig 3C). The enzymatic assays confirm the results from the yeast assays showing that M^pro^ E166R, E166A, and E166V are resistant to nirmatrelvir and also show that there is cross-resistance to PF-0085231 and GC-376 (Figs 3B, 3C and S6C). Similarly, results from yeast assays of M^pro^ N142A mutant appears to correspond well to the *in vitro* assays as both show minor to no increases in resistance (Fig 3B and 3C). Furthermore, the M^pro^ E166N and E166D mutants, which is not predicted to be catalytically active from the yeast assay, displayed >70-fold decreases in activity compared to wild-type in the *in vitro* assays. This result is completely consistent with observing no growth reduction when expressed in yeast. Taken together there is good correlation between the enzyme and yeast assays.

### Crystal structure of M^pro^ E166R reveals a loss of interactions leading to drug resistance

**Fig 4:**
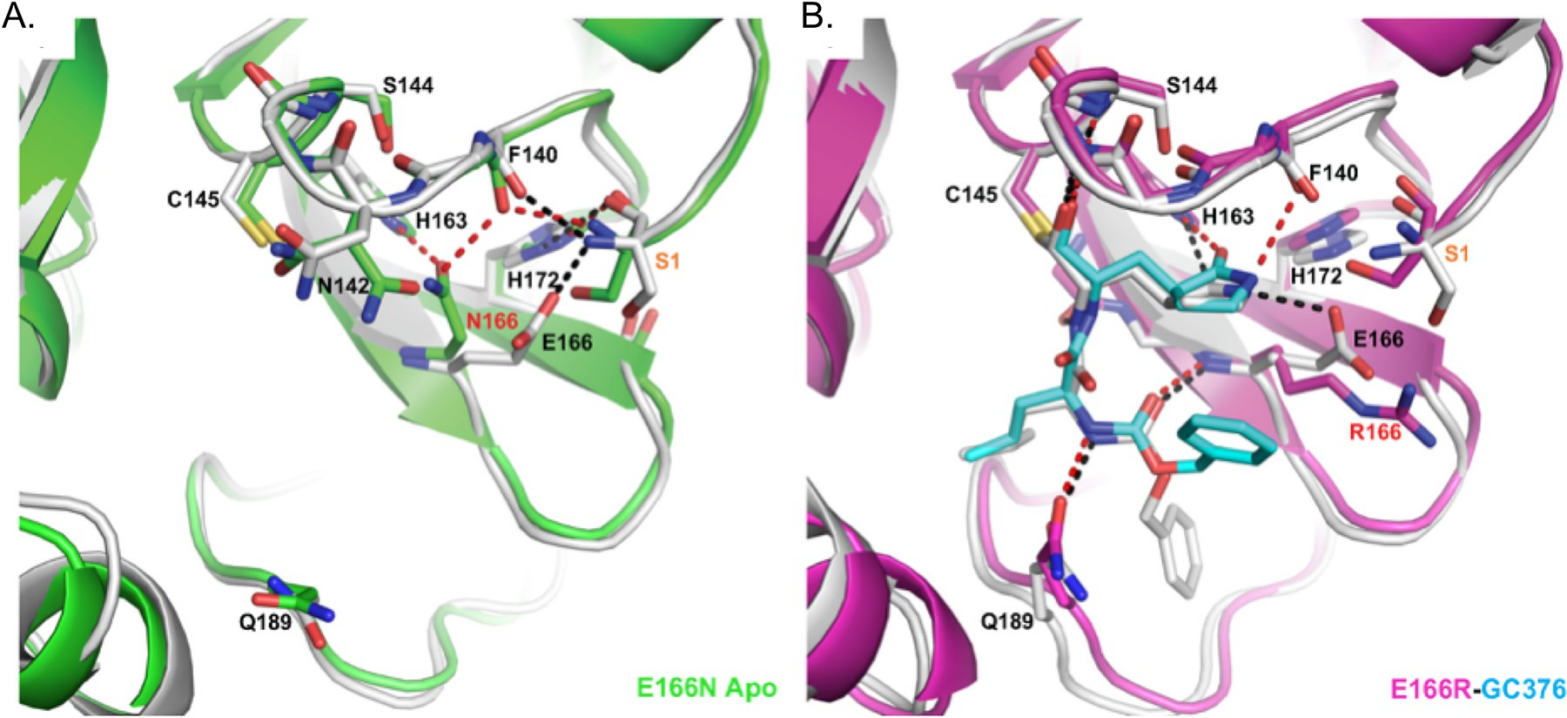
Crystal structures reveals structural basis for E166R resistance and E166N inactivity. A) Apo M^pro^ WT (white, PDB 7JP1) aligned with apo M^pro^ E166N (green, PDB 8DDI). B) M^pro^ WT GC-376 complex (white, PDB 6WTT) aligned with M^pro^ E166R GC-376 complex (magenta, PDB 8DDM). WT hydrogen bonds are shown as black dashes, and mutant hydrogen bonds are shown as red dashes. GC-376 is shown in white for the WT structure and cyan for the mutant structure. Mutations are indicated with red text. Ser1 from an adjacent protomer is indicated with orange text.

We were particularly interested in how replacing glutamate at position 166 with arginine led to a >1000-fold increase in resistance to nirmatrelvir while a substitution with asparagine led to an 83.5-fold decrease in enzymatic activity even though E166 is not known to be directly involved in catalysis. Toward addressing both questions, we solved the crystal structure of apo M^pro^ E166N and the complex structure of M^pro^ E166R with GC-376 at 2.3 and 2.1 Å resolution, respectively (Fig 4). Both proteins were crystallized in the C2 space group with one M^pro^ molecule per asymmetric unit, and the biological dimer can be generated through crystallographic symmetry. The conformations of the protein and ligand are therefore exactly the same between the two protomers of the dimer in these structures. Our efforts to obtain nirmatrelvir complex failed due to the relatively low compound solubility and the reduced binding for the mutant. But both nirmatrelvir and GC-376 have the same pyrrolidone side chain placed in the S1 site where E166 is located. In previous WT complex structures (PDB codes, 7RFW (nirmatrelvir), 6WTT(GC-376)), the protein and the pyrrolidone side chain adopted identical conformations in this area. The GC-376 complex can thus offer important insights into how E166 mutations may impact the binding of ligands with similar chemical structures, including nirmatrelvir.

In the M^pro^ E166N mutant structure, N166 forms a hydrogen bond (HB) with H163, an interaction not observed between E166 and H163 in the wild-type M^pro^ structure (Fig 4A). This new HB prevents H163 from hydrogen bonding with the glutamine side chain of the substrate, an interaction crucial to substrate binding. The binding of the substrate would therefore require N166 to adopt a different conformation, breaking the HB with H163 and increasing the energetic cost. These observations are consistent with the drastic decrease of activity in the E166N mutant and lack of toxicity when expressed in yeast (Fig 2C) bringing to light how residues outside of the catalytic core can influence substrate binding.

In contrast, the longer and positively charged R166 side chain in the M^pro^ E166R mutant does not interact with H163, but rather extends into the solvent (Fig 4B). Therefore, the S1 site is open for substrate binding. However, the E166R mutation does affect ligand binding in several aspects. The negatively charged E166 side chain forms two crucial HBs, one with the N-terminus of the neighboring M^pro^ protomer in the biological dimer, and the other with the pyrrolidone side chain of inhibitors (in both nirmatrelvir and GC-376) or with the glutamine side chain of the substrate as described above. The E166R mutation would abolish this direct HB with the substrate or inhibitor, resulting in the pyrrolidone ring of GC-376 forming an alternative weak HB with F140 backbone carbonyl group (3.1 Å in length) in the mutant complex structure (Fig 4B). In addition, the N-terminus of the enzyme interacts with both E166 and the backbone carbonyl group of F140, and plays an important role in maintaining the structural stability of the enzyme active site. The E166R mutation eliminates the salt bridge with the N-terminus of the adjacent protomer, and further introduces electrostatic repulsion leading to small yet significant changes in the N-terminus conformation. Consequently, the distance between the N-terminal amine group and the F140 carbonyl group increased from 2.6 Å in the WT to 3.7 Å in the M^pro^ E166R mutant, diminishing the HB. This in turn may destabilize the loop that F140 resides on and also contains other important structural features involved in enzyme catalysis and ligand binding, including the backbone amide groups of Gly143 and Ser144 that form part of the oxyanion hole to stabilize the reaction transition state. This loop also contains the peptide bond between Leu141 and Asn142 that interacts with the two extra carbon atoms of the inhibitor pyrrolidone ring, but not with the substrate glutamine side chain. Destabilization of the region near F140 may increase the entropic cost of binding to the rigid pyrrolidone ring of nirmatrelvir and GC-376, more than the smaller and more flexible substrate glutamine side chain. For similar entropic reasons, the HB between the pyrrolidone ring and E166 might contribute more to inhibitor binding than that between the more flexible glutamine side chain and E166 (Fig 4B). Consequently, the E166R mutation may have a stronger effect on binding to inhibitors such as nirmatrelvir versus substrate.

## Discussion

In sum, we demonstrate that using yeast growth as an approximation for M^pro^ activity can be a reliable indicator of the effects that mutations in M^pro^ can have on its activity and potential for drug resistance. Yeast assays indicated that E166 substituted with R, V, and A were all resistant to nirmatrelvir and *in vitro* enzyme assays confirmed this observation, revealing a ~47 to ~5000-fold increase in Ki. Furthermore, the C145A catalytic mutant and E166N and E166D mutants did not cause a growth reduction in yeast and enzyme assays showed that the E166D or N substitution confers a dramatic ~74- or ~84-fold decrease in activity, respectively. In yeast assays the N142A mutant displayed minor differences in drug sensitivity compared to wild-type (RC_50_ ~1.5-fold more than WT), which was confirmed by our *in vitro* enzyme assays. Similarly, the P132H mutant remained sensitive to nirmatrelvir based on our yeast assay, potentially even more sensitive with an RC_50_ ~2.8-fold less than WT. This is consistent with previous reports showing that the P132H mutant remains sensitive to nirmatrelvir in *in vitro* enzyme assays [29–32]. It appears that M^pro^ mutants (i.e. E166R) that have a decrease in catalytic efficiencies of up to 16-fold compared to WT are still able to confer a marked reduction in yeast growth. This is important as resistant mutants are likely to reduce protein fitness [33,34]. However, the yeast assay is unable to detect enhanced M^pro^ activity (e.g., M^pro^ N142A), which we observed in *in vitro* assays. This may have been due to the relatively small increase (1.4-fold). However, the enhanced activity associated with N142A suggests that M^pro^ can evolve to be a more active enzyme. It is possible that mutants which enhance M^pro^ activity can improve protein fitness when combined with resistant mutants that on their own may have reduced activity [15]. Recent reports from in vitro evolution experiments suggest that single mutants such as E166A and E166V, which do provide resistance, have significant reductions in proteolytic activity and require compensatory mutations to improve fitness levels necessary for infection at least in vitro [14,16,17]. While the yeast system does not identify the necessary compensatory mutation(s), it nonetheless can point to mutations that confer resistance and to prioritize mutants that are of interest for further studies. The crystal structure of E166R with GC-376 revealed loss of key hydrogen bonds with the pyrrolidone ring of GC-376 which can explain the increase in resistance to nirmatrelvir containing the same functional group. The E166A and E166V mutations would have a similar effect by abolishing the hydrogen bonds involving the E166 side chain, therefore reducing direct contacts with the substrate/inhibitor and destabilizing the active site. On the other hand, the E166N mutant which could be considered a more conserved change than E166R decreased activity by ~83-fold and did not confer a growth reduction in the yeast assays. In turn the crystal structure shows that the asparagine prevents substrate binding through a new hydrogen bond with H163, providing a mechanism to explain the significant reduction in activity. The additional mutants at E166 that are associated with *in vitro* viral evolution experiments along with what we show here highlight the importance of this site in playing a role in nirmatrelvir resistance. Our crystal structure illuminates a structural mechanism to help explain how substitutions at E166 can either lead to loss of activity versus gain of resistance.

While the drug doses used with yeast are in the micromolar versus nanomolar range that is more typical of *in vitro* enzymatic or viral assays, we observed good correlations between the yeast and enzymatic assays for nearly all of the mutants tested. The higher concentrations of drug may be needed even though we deleted the major efflux pump, Pdr5, as yeast harbor a range of efflux activities [35], or possibly differences in permeability as a result of lipid composition differences from human cells, as well as potential drug interactions with the yeast cell wall [36]. Additional differences observed between the yeast and enzymatic assays may be a result of having multiple substrates in yeast, additional complexity of the cellular proteome, differences in pH, salt, and oxidation levels. Analysis of the top 153 proteins (p<0.05) with reduced abundance relative to strains expressing M^pro^ C145A revealed that 16% (25) have at least one M^pro^ site (LQ/S, LQ/A, or LQ/G) (S2 Table) suggesting that these may be direct M^pro^ substrates [22,23]. There were another 36% (55) of proteins that have non-canonical M^pro^ sites in which LQ is followed by any amino acid besides S, A, or G. A few reports identifying human host substrates suggest that M^pro^ might recognize such sites [37,38]. Thus, the total possible substrates may be greater than 16% of the total proteins we found to be reduced when M^pro^ is present. However, it is likely that these 36% of proteins are reduced due to a secondary consequence of the primary yeast substrates with canonical sites. We acknowledge that this may also suggest that drug resistance phenotypes in yeast may in some cases not directly indicate resistance relative to the viral cleavage sites. However, finding mutants (e.g. E166A and E166V) known to be compatible with virus replication and drug resistance to also display resistance in yeast suggests that it can be a good proxy for mutants of interest.

Taken together, these results demonstrate that a non-pathogenic, rapid, inexpensive and highly accessible yeast-based method can be used to characterize mutants for both their effects on M^pro^ activity and their responses to inhibitor compounds. There are reports using yeast as a tool to screen for M^pro^ inhibitors or that use deep mutational scanning of M^pro^ to identify high and low tolerant sites [16,39,40]. Unlike these previous reports we show that nirmatrelvir resistant mutants can be tested directly in yeast using growth as an approximation for M^pro^ drug resistance. The qualitative results from the yeast assays can be an important tool to help prioritize mutants of interest before moving ahead to more demanding viral based experiments. As more inhibitors are used in the general population there will be increasing selection pressures for drug resistant mutations that will go beyond the current set of mutants that are potentially drug resistant [41,42]. The yeast system reported here promises to be an invaluable tool in helping to combat future drug resistant mutations to stem the tide of COVID-19 infections.

## Materials and methods

### Strains, media, and chemicals

All yeast strains carried a *pdr5*::*G418* deletion in the BY4741 background (*MATa his3Δ1 leu2Δ0 met15Δ0 ura3Δ0*). Yeast were grown in liquid synthetic complete (SC) media (0.17% yeast nitrogen base, 0.5% ammonium sulfate, amino acid mix with appropriate drop out as noted, 2% glucose) or on solid SC media containing 2% agar at 30°C. Media and reagents for culturing yeast were from United States Biological (Salem, MA). M^pro^ and PL^pro^ inhibitors were from MedChemExpress (Monmouth Junction, NJ) and Selleck Chemicals (Houston, TX). All other chemicals were from Sigma Aldrich (St. Louis, MO) or VWR (Radnor, PA).

### Expression of SARS-CoV-2 genes in yeast and mutagenesis

The indicated SARS-CoV-2 genes were codon optimized for yeast, tagged at the 3’ with a 3X-Flag epitope, carried on high copy plasmids and genes were under the control of the Gal1 promoter (see S3 Table). Site directed mutagenesis was performed using In-Fusion Cloning Kit (Takara). Primers used for mutagenesis can be found in S2 Table. The M^pro^ gene was sequenced to confirm that mutations were incorporated successfully.

### Yeast transformation

A single yeast colony was used to inoculate 5ml liquid YPD (1% yeast extract, 1% yeast bacto-peptone, 2% glucose) and grown overnight at 30°C. The next day cells were washed and resuspended in 1ml lithium acetate/TE solution (100 m*M* lithium acetate, 10 m*M* Tris-HCl, 1 m*M* EDTA, pH 7.5). Cells were aliquoted (60 μl) into microcentrifuge tubes, followed by the addition of denatured salmon sperm DNA (50μg), 0.2μg of plasmid, 1ml polyethylene glycol (PEG) lithium acetate solution (40% (w/v) PEG 4000, 100 m*M* lithium acetate, 10 m*M* Tris-HCl, 1 m*M* EDTA, pH 7.5), and incubated for 45min at 30°C. This was followed by a 20min incubation at 42° and chilled for 2min on ice. Cells were washed and resuspended in 100μl H_2_O and plated on selective SC agar plates, incubated for ~3 days at 30°C.

### Protein extraction and western analysis

Cells were grown overnight in 5 ml SC-Ura, 2% raffinose at 30°C. The next day, fresh cultures were started with optical density OD_600_ of 0.5 in 20 ml SC-Ura, 2% galactose at 30° for 6 hrs. Cells were then harvested, frozen in liquid nitrogen, and stored at −80°. For total protein extract, trichloroacetic acid was performed as described previously [43] and protein concentration was determined by BCA protein assay kit (Thermo scientific). Protein samples were separated by 4–12% gradient SDS-PAGE (GenScript) and blotted onto nitrocellulose or PVDF membranes. The following primary antibodies were used at 1:5000 dilution: anti-FLAG antibody (GenScript), and anti-GAPDH antibody (Proteintech). Secondary anti-mouse IgG HRP antibody was used at 1:7000 dilution (Promega). ChemiDoc (Bio-Rad) imaging system was used to detect chemiluminescence signals from western blots. Relative quantification of protein bands (α-Flag, α-GAPDH) from western blots was performed using Fiji [44].

### Cell growth assays and RC_50_ measurements

Cells were grown overnight in 5ml SC-Ura, 2% raffinose at 30°C. The next day, fresh cultures were started with an OD_600_ of 0.1 in SC-Ura, 2% galactose, with or without inhibitors and transferred to to 96-well plates, incubated at 30°C on a a rotary shaker. Three independent transformants were used to test each form of M^pro^. Each transformant was sampled three times for each assay. The plate was transferred to a Tecan Infinite 200 PRO plate reader, and OD_600_ measurements were taken at 0, 24, 48 and 72 hours, with 5 flashes per well. Excel (Microsoft) was used to analyze the raw data. As a measure of inhibitory activity of nirmatrelvir we calculated a Recovery Concentration (RC_50_). The slopes from the dose responses were calculated and used to estimate the concentration of inhibitor that improves growth to half-maximal relative to empty vector control after 72 hours of growth. As we only tested up to 200μM of nirmatrelvir, in cases where the RC_50_ is beyond this concentration we indicate as >200μM.

### Yeast proteomics

Cells were grown overnight in 10ml SC-Ura + 2% Raffinose media at 30°C. The next day, fresh cultures were started with OD_600_ of 0.1 in 100ml SC-Ura + 2% galactose at 30°C for 6 hr. Cells were then harvested, frozen in liquid nitrogen, and stored at −80°. Protein extraction was performed as described previously [43] and protein concentration was determined using Pierce BCA protein assay kit (Thermo scientific).

To determine changes in the proteome associated with expression of M^pro^ versus M^pro^ C145A, in-solution tryptic digestion was performed as described [45] followed by desalting with a Pierce Peptide Desalting Spin Columns per the manufacturer’s protocol (ThermoFisher Scientific, cat no. 89852) and the peptides were dried by vacuum centrifugation. 600 ng of the final sample was analyzed by mass spectrometry. HPLC-ESI-MS/MS was performed as previously described [46]. In brief, MS/MS was performed in positive ion mode on a Thermo Scientific Orbitrap Fusion Lumos tribrid mass spectrometer fitted with an EASY-Spray Source (Thermo Scientific, San Jose, CA). NanoLC was performed using a Thermo Scientific UltiMate 3000 RSLCnano System with an EASY Spray C18 LC column (Thermo Scientific).

Tandem mass spectra were extracted from Xcalibur ‘RAW’ files and charge states were assigned using the ProteoWizard 2.1.x msConvert script using the default parameters(23). The fragment mass spectra were then searched against the *Saccharomyces cerevisiae* (strain ATCC 204508 / S288c) (Baker’s yeast) UniProt database (6067 entries) using Mascot (Matrix Science, London, UK; version 2.6) using the default probability cut-off score. Cross-correlation of Mascot search results with X! Tandem was accomplished with Scaffold (version Scaffold_4.8.7; Proteome Software, Portland, OR, USA). Probability assessment of peptide assignments and protein identifications were made through the use of Scaffold. Only peptides with ≥ 95% probability were considered. Progenesis QI for proteomics software (version 2.4, Nonlinear Dynamics Ltd., Newcastle upon Tyne, UK) was used to perform ion-intensity based label-free quantification similar to as previously described [46]. Principal component analysis and unbiased hierarchal clustering analysis (heat map) was performed in Perseus [47,48]. Gene ontology and KEGG pathway enrichment analysis was performed with DAVID [49]. Proteomics data has been deposited to ProteomeXchange. Project accession: PXD036325 and Project DOI:10.6019/PXD036325.

### Recombinant M^pro^ and proteolytic activity assays

SARS-CoV-2 M^pro^ mutants were generated with QuikChange II Site-Directed Mutagenesis Kit from Agilent (Catalog #200524), using plasmid pE-SUMO-M^pro^ as the template. The plasmid produces tag-free M^pro^ protein with no extra residue at either N- or C-terminus upon removal of the SUMO tag by SUMO protease digestion [21].

SARS-CoV-2 M^pro^ mutant proteins were expressed and purified as previously described [21,50] with minor modifications. Plasmids were transformed into E. coli BL21(DE3) competent cells and bacterial cultures overexpressing the target proteins were grown in LB (Luria-Bertani) medium containing 50 μg/mL of kanamycin at 37°C, and expression of the target protein was induced at an optical density (A600) of 0.6–0.8 by the addition of isopropyl β-d-1-thiogalactopyranoside (IPTG) to a final concentration of 0.5 mM. The cell culture was incubated at 18°C for 12–16 hrs. Bacterial cultures were harvested by centrifugation (8,000 ×g, 10 min, 4°C) and resuspended in lysis buffer containing 25 mM Tris (pH 8.0), 750 mM NaCl, 2 mM DTT, 0.5 mg/mL lysozyme, 0.5 mM phenylmethylsulfonyl fluoride (PMSF) and 0.02 mg/mL DNase I. Bacterial cells were lysed by alternating sonication (30% amplitude, 1 s on/1 s off) and homogenization using a tissue grinder. The lysed cell suspension was clarified by centrifugation (18,000 ×g, 30min, 4°C) and the supernatant was incubated with Ni-NTA resin for over 2 hrs at 4°C on a rotator. The Ni-NTA resin was thoroughly washed with 20 mM imidazole in washing buffer containing 50mM Tris (pH 8.0), 150mM NaCl, 2 mM DTT, and SUMO-M^pro^ protein was eluted with elution buffer containing 50 to 300mM imidazole, 50mM Tris (pH 8.0), 150mM NaCl, 2mM DTT. Fractions containing SUMO-M^pro^ proteins greater than 90% homogeneity were pooled and subjected to dialysis (two times) against a buffer containing 50mM Tris (pH 8.0), 150mM NaCl, 2mM DTT and 10% glycerol. SUMO protease digestion was carried out at 30°C for 1 hr to remove SUMO tag. Following digestion, SUMO Protease and SUMO tag were removed by Ni-NTA resin. The purified tag-free SARS-CoV-2 M^pro^ mutant proteins were fast frozen in liquid nitrogen and stored at -80°C.

For measurement of *K*_*m*_*/V*_*max*_ of SARS-CoV-2 M^pro^ mutants, proteolytic reactions were carried out with optimized concentrations of the mutant proteins and a series of concentrations of FRET substrate, Dabcyl-KTSAVLQ/SGFRKME (Edans), ranging from 0 to 200 μM in 100μL of reaction buffer containing 20mM HEPES (pH 6.5), 120mM NaCl, 0.4mM EDTA, 4mM DTT, and 20% glycerol at 30°C in a BioTek Cytation 5 imaging reader (Agilent) with filters for excitation at 360/40 nm and emission at 460/40 nm. The SARS-CoV-2 M^pro^ FRET substrate used in this study is Dabcyl-KTSAVLQ/SGFRKME (Edans), which corresponds to the sequence between viral polypeptide NSP4-NSP5 junction from SARS-CoV-2 [21,51]. Reactions were monitored every 90s, and the initial velocity of the proteolytic activity was calculated by linear regression for the first 15min of the kinetic progress curves. The initial velocity was plotted against the FRET substrate concentrations using the classic Michaelis-Menten equation in Prism 8 software.

For IC_50_ measurements, optimized concentrations of the mutant proteins were incubated with series concentrations of GC-376, PF-00835231 or nirmatrelvir (PF-07321332) in 100μL of reaction buffer at 30°C for 15 min, and the reaction was initiated by adding 10μM FRET substrate. The reaction was monitored for 1 hr, and the initial velocity was calculated for the first 15min by linear regression. The IC_50_ was determined by plotting the initial velocity against various concentrations of the compounds using log (inhibitor) vs response-variable slope in Prism 8 software.

For *K*_*i*_ measurements, optimized concentrations of the mutant proteins were added to 20μM FRET substrate with various concentrations of GC-376, PF-00835231 or nirmatrelvir (PF-07321332) in 200μL of reaction buffer at 30°C to initiate the proteolytic reaction. The reaction was monitored for 2 hrs and the initial velocity was calculated for the first 90 min by linear regression. The *K*_*i*_ was calculated by plotting the initial velocity against various concentrations of the compounds using Morrison plot (tight binding) in Prism 8 software.

### M^pro^ crystallization and structure determination

SARS-CoV-2 M^pro^ E166N/R was diluted to 5 mg/mL in protein buffer (50 mM Tris pH 7.0, 150 mM NaCl, 4 mM DTT). Protein for complex determination was incubated overnight at 4°C with 2mM GC-376. No precipitation was observed after incubation, and centrifugation was not necessary. Apo and complex crystals were grown using 1.5 μL:1.5 μL (protein:well solution) hanging drops and a well solution of 0.1 M MgCl_2_, 20% PEG 3350, 10% 1,6-hexanediol, 0.1 M HEPES pH 7.5, and 0.1 M LiSO_4_. E166N/R crystals grew overnight at 20°C. Crystals were cryoprotected using the well solution supplemented with 20% glycerol, and then flash-frozen in liquid nitrogen.

X-ray diffraction data (S4 Table) were collected at the Southeast Regional Collaborative Access Team (SER-CAT) 22-BM beamline at the Advanced Photon Source (APS) in Argonne, IL, and processed with HKL2000 and CCP4. PHASER was used for molecular replacement using a previously solved SARS-CoV-2 M^pro^ structure (PDB ID: 7LYH) as a reference model. The CCP4 suite, (23) Coot, (24) and the PDB REDO server (pdb-redo.eu) (25) were used to complete the model building and refinement. The PyMOL Molecular Graphics System (Schrödinger, LLC) was used to generate all images.

## Supporting information

S1 Table List of yeast proteins that displayed significant changes in abundance when the catalytic mutant M^pro^ was expressed versus wild-tye M^pro^. (XLSX)

S2 Table Presence or absence of canonical M^pro^ cleavage site(s) in the top 153 proteins (p<0.05) with reduced abundance in strains expressing wild-type M^pro^. (XLSX)

S3 Table Plasmids and primers used in this study. (DOCX)

S4 Table X-ray Data Collection and Refinement Statistics Data Collection. (DOCX)

S1 Fig Expression of SARS-CoV-2 Genes in yeast. A) SARS-CoV-2 genes were cloned into a high copy yeast plasmid and regulated by a galactose inducible promoter (Gal1). Growth curve of yeast carrying EV, M^pro^ WT, or M^pro^ C145A in glucose (Glu) versus galactose (Gal). B) Spot assays were performed on glucose (left) or galactose (right) containing plates to determine the growth effects conferred by expression of the indicated genes. Two biological replicates were performed for each (two rows) EV and indicated gene. Samples (3ul) of a 5-fold serial dilution are spotted in each row. EV refers to yeast carrying an empty vector. M, E, S, N are structural proteins and all others are non-structural proteins (NSP).
(TIFF)

S2 Fig Determining potential yeast substrates of M^pro^. Schematic of the work flow to perform proteomics from yeast strains expressing wild-type M^pro^ or catalytically inactive M^pro^ C145A.
(TIFF)

S3 Fig Effects of nirmatrelvir on yeast expressing M^pro^ from SARS-CoV-1 and Bat-CoV-HKU9. Growth of yeast expressing M^pro^ from the indicated coronavirus is plotted in the presence or absence of nirmatrelvir. M^pro^ from both viruses remain responsive to nirmatrelvir as indicated by improved growth (nearly matching the empty vector (EV) control) in the presence of nirmatrelvir. (*, p<0.01; ***, p<0.001) indicates differences compared to EV.
(TIFF)

S4 Fig Growth curves for yeast expressing SARS-CoV-2 M^pro^. A) Indicated M^pro^ mutants were expressed in yeast. Wild-type M^pro^, M^pro^ E166R, M^pro^ P132H, and M^pro^ N142A all confer a strong growth defect compared to empty vector (EV) control. On the other hand M^pro^ E166N and M^pro^ E166D do not confer a growth defect and grow as well as EV control. B-D) Growth curves for M^pro^ E166R, M^pro^ P132H, and M^pro^ N142A treated with 0, 25, 50, 100, 200mM of nirmatrelvir. E) Western blot showing levels of M^pro^ WT, E166R, and N142A.
(TIFF)

S5 Fig Yeast growth assays show M^pro^ mutants E166A and E166V are resistant to nirmatrelvir. A) Total growth of cultures after 72 hours expressing M^pro^ WT, E166A, and E166V. B) Western analysis shows that mutants and wild-type M^pro^ are expressed at comparable levels. C-D) E166A and E166V appear to be resistant as there is a lack of growth even when treated with 200mM of nirmatrelvir. (***) indicates differences (p<0.001) compared to EV. Ratios of Flag:GAPDH signals relative to M^pro^ WT is shown at the bottom of each lane.
(TIFF)

S6 Fig Enzymatic assays demonstrate that E166A and E166V are resistant to nirmatrelvir and other M^pro^ inhibitors. A) Michaelis–Menten plot of M^pro^ and its mutants with various concentrations of FRET substrate. The Km, Vmax, kcat, and kcat/Km values are shown in the table. B) The IC50 plots of nirmatrelvir against M^pro^ E166A and E166V. C) Ki plots of nirmatrelvir, GC-376, and PF-00835231 against M^pro^ E166A and E166V.
(TIFF)
