# Supplementary material for: A yeast-based system to study SARS-CoV-2 Mpro structure and to identify nirmatrelvir resistant mutations
Source: PLoS Pathog. 2023 Aug 31;19(8):e1011592. doi: 10.1371/journal.ppat.1011592 (PMC10499260; doi:10.1371/journal.ppat.1011592)
Supplement: S3 Table — (DOCX) [file ppat.1011592.s003.docx]

S3 Table. Plasmids and primers used in this study

| Number | ^1^Plasmid | Gene |
| --- | --- | --- |
| JCB462 | pGBW-m4046203 | SARS-CoV-2 M^pro^ (3CL^pro^) |
| JCB464 | pGBW-m4046418 | SARS-CoV-2 N (Nucleocapsid) |
| JCB465 | pGBW-m4046249 | SARS-CoV-2 NSP12 (RNA-dependent RNAP) |
| JCB467 | pGBW-m4046574 | SARS-CoV-2 S (Spike protein) |
| JCB504 | pGBW-m4046277 | SARS-CoV-2 M (Membrane protein) |
| JCB505 | pGBW-m4046231 | SARS-CoV-2 NSP8 |
| JCB506 | pGBW-m4046455 | SARS-CoV-2 E (Envelope protein) |
| JCB507 | pGBW-m4046246 | SARS-CoV-2 NSP7 |
| JCB508 | pGBW-m4046415 | SARS-CoV-2 NSP13 (Helicase) |
| JCB509 | pGBW-m4046483 | SARS-CoV-2 NSP3 (PL^pro^) |

^1^ Plasmids were gifts from Ginkgo Bioworks & Benjie Chen and obtained from Addgene.

| Mutant | F or R | Sequence |
| --- | --- | --- |
| C145A | Forward Primer | 5'-GGTTCTGCTGGCTCCGTTGGTTTTA-3' |
|  | Reverse Primer | 5'-GGAGCCAGCAGAACCATTCAAAAAA-3' |
| E166A | Forward Primer | 5'-TCACATGGCTTTGCCTACAGGTGTTCACGC-3' |
|  | Reverse Primer | 5'-GGCAAAGCCATGTGATGCATGTAACAGAAACTG-3' |
| E166V | Forward Primer | 5'-TCACATGGTTTTGCCTACAGGTGTTCACGC-3' |
|  | Reverse Primer | 5'-GGCAAAACCATGTGATGCATGTAACAGAAACTG-3' |
| E166R | Forward Primer | 5'-TCACATGAGATTGCCTACAGGTGTTCACGC-3' |
|  | Reverse Primer | 5'-GGCAATCTCATGTGATGCATGTAACAGAAACTG-3' |
| E166N | Forward Primer | 5'-TCACATGAACTTGCCTACAGGTGTTCACGC-3' |
|  | Reverse Primer | 5'-GGCAAGTTCATGTGATGCATGTAACAGAAACTG-3' |
| E166D | Forward Primer | 5'-TCACATGGACTTGCCTACAGGTGTTCACGC-3' |
|  | Reverse Primer | 5'-GGCAAGTCCATGTGATGCATGTAACAGAAACTG-3' |
| P132H | Forward Primer | 5'-TATGAGGCACAACTTCACTATTAAGGGTTCTTTTT-3' |
|  | Reverse Primer | 5'-AAGTTGTGCCTCATAGCGCACTGGTATACACC-3' |
| N142A | Forward Primer | 5'-TTTTTTGGCTGGTTCTTGTGGCTCCGTT-3' |
|  | Reverse Primer | 5'- GAACCAGCCAAAAAAGAACCCTTAATAGTGAAG-3' |
