# Supplementary material for: A yeast-based system to study SARS-CoV-2 Mpro structure and to identify nirmatrelvir resistant mutations
Source: PLoS Pathog. 2023 Aug 31;19(8):e1011592. doi: 10.1371/journal.ppat.1011592 (PMC10499260; doi:10.1371/journal.ppat.1011592)
Supplement: S4 Table — (DOCX) [file ppat.1011592.s004.docx]

S4 Table. X-ray Data Collection and Refinement Statistics

Data Collection
