# Supplementary figures and images for: A yeast-based system to study SARS-CoV-2 Mpro structure and to identify nirmatrelvir resistant mutations
Source: PLoS Pathog. 2023 Aug 31;19(8):e1011592. doi: 10.1371/journal.ppat.1011592 (PMC10499260; doi:10.1371/journal.ppat.1011592)

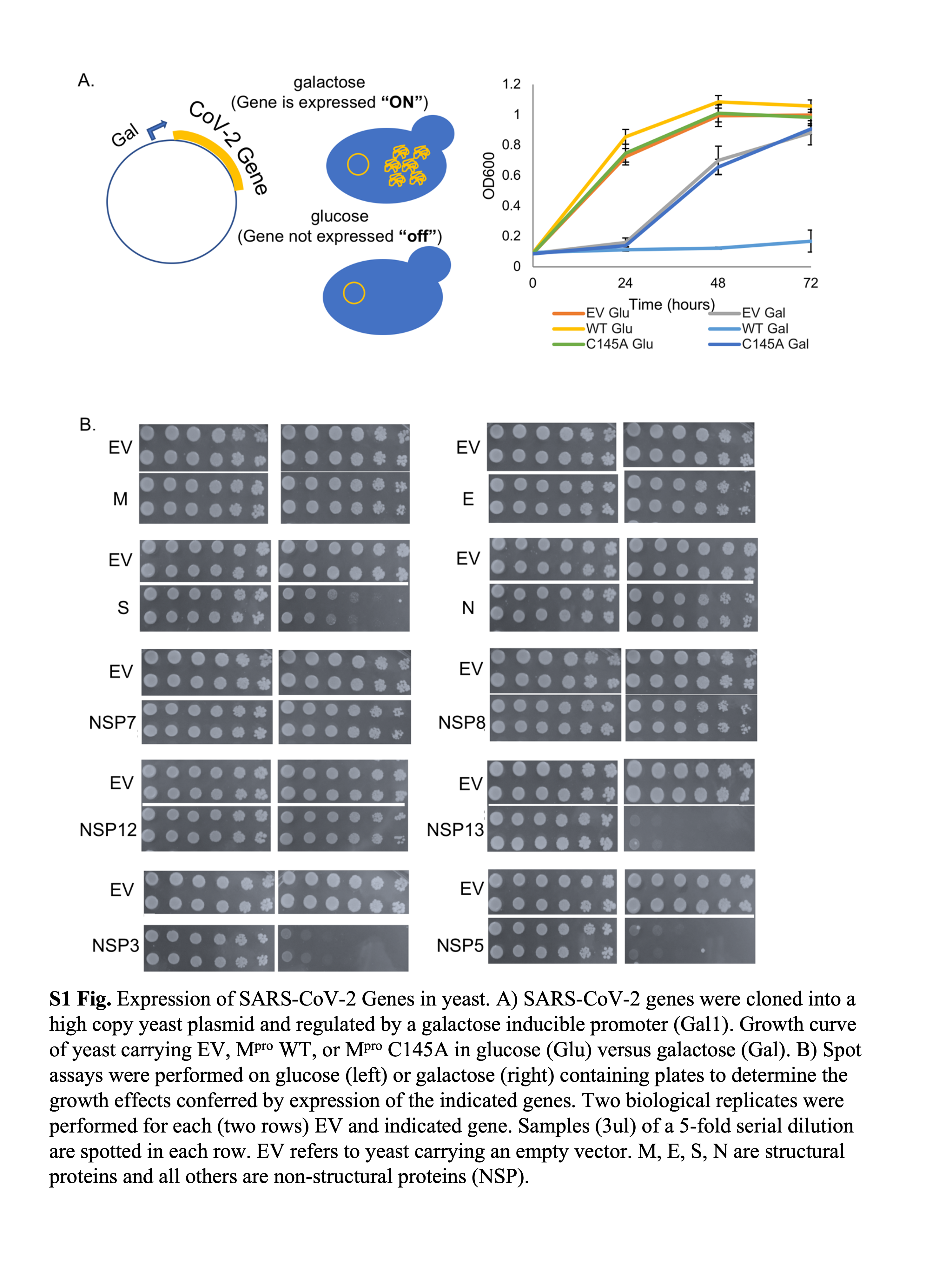

Supplement: S1 Fig — A) SARS-CoV-2 genes were cloned into a high copy yeast plasmid and regulated by a galactose inducible promoter (Gal1). Growth curve of yeast carrying EV, Mpro WT, or Mpro C145A in glucose (Glu) versus galactose (Gal). B) Spot assays were performed on glucose (left) or galactose (right) containing plates to determine the growth effects conferred by expression of the indicated genes. Two biological replicates were performed for each (two rows) EV and indicated gene. Samples (3ul) of a 5-fold serial dilution are spotted in each row. EV refers to yeast carrying an empty vector. M, E, S, N are structural proteins and all others are non-structural proteins (NSP). (TIFF) [file ppat.1011592.s005.tiff]

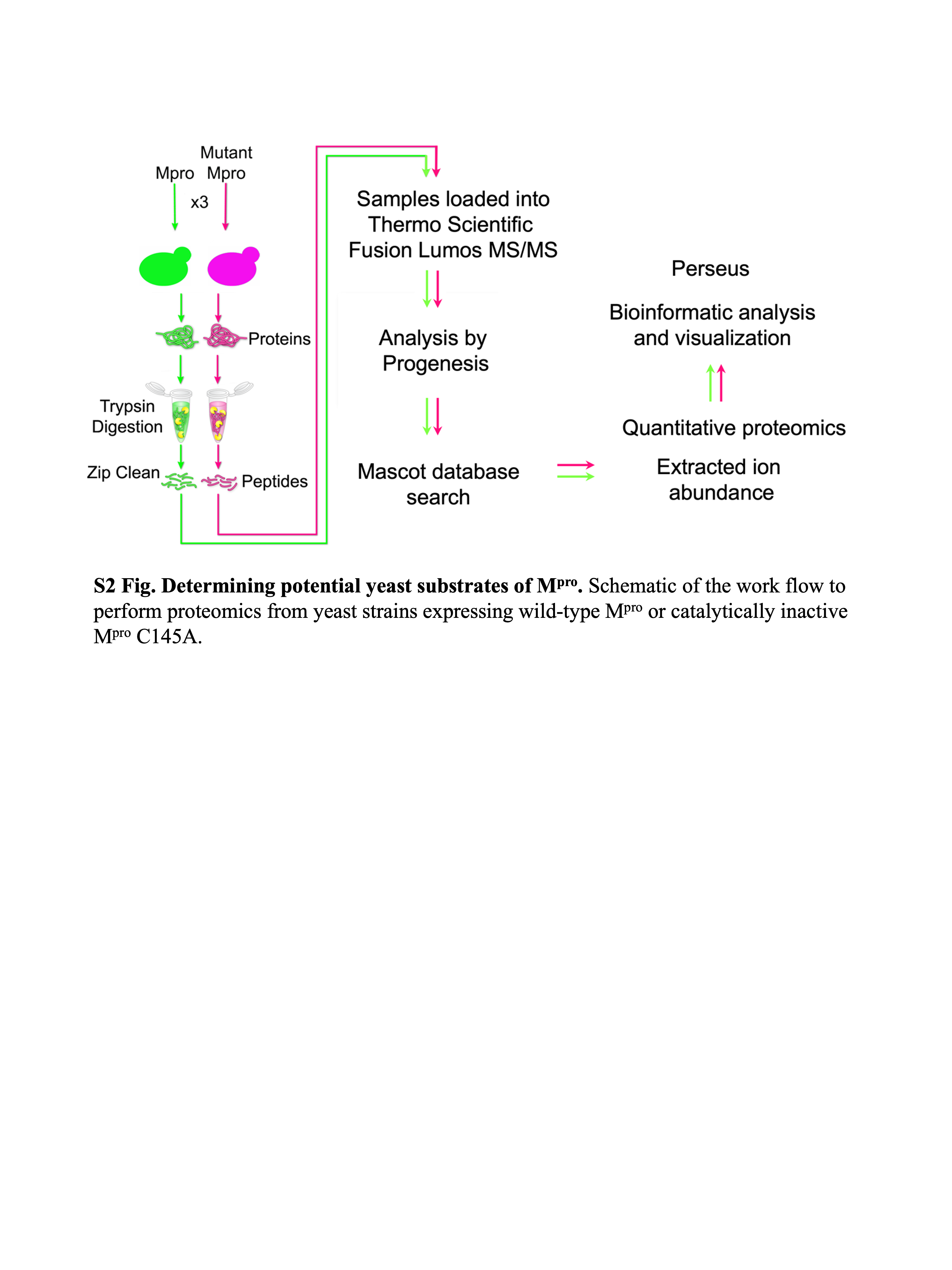

Supplement: S2 Fig — Schematic of the work flow to perform proteomics from yeast strains expressing wild-type Mpro or catalytically inactive Mpro C145A. (TIFF) [file ppat.1011592.s006.tiff]

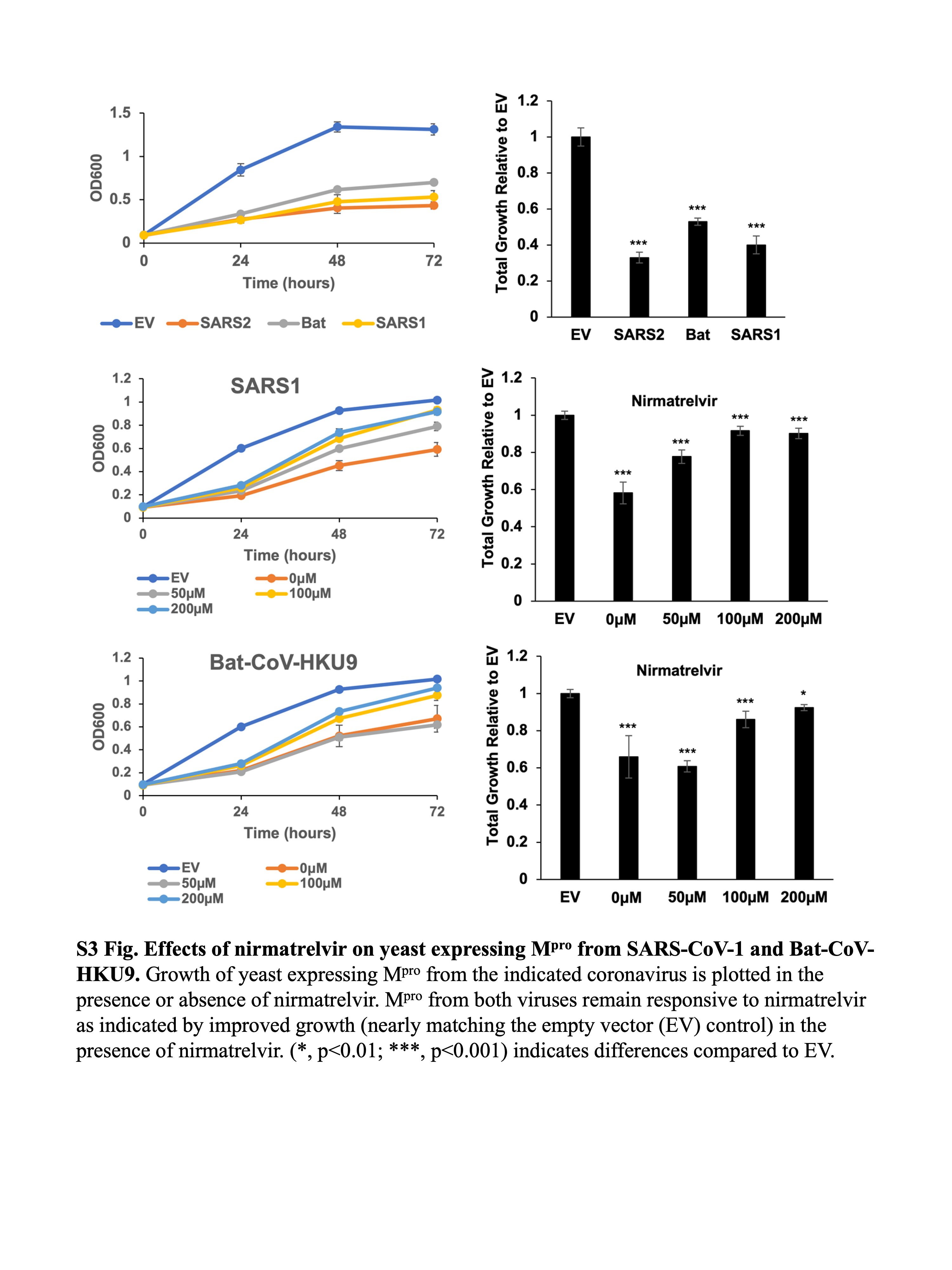

Supplement: S3 Fig — Growth of yeast expressing Mpro from the indicated coronavirus is plotted in the presence or absence of nirmatrelvir. Mpro from both viruses remain responsive to nirmatrelvir as indicated by improved growth (nearly matching the empty vector (EV) control) in the presence of nirmatrelvir. (*, p<0.01; ***, p<0.001) indicates differences compared to EV. (TIFF) [file ppat.1011592.s007.tiff]

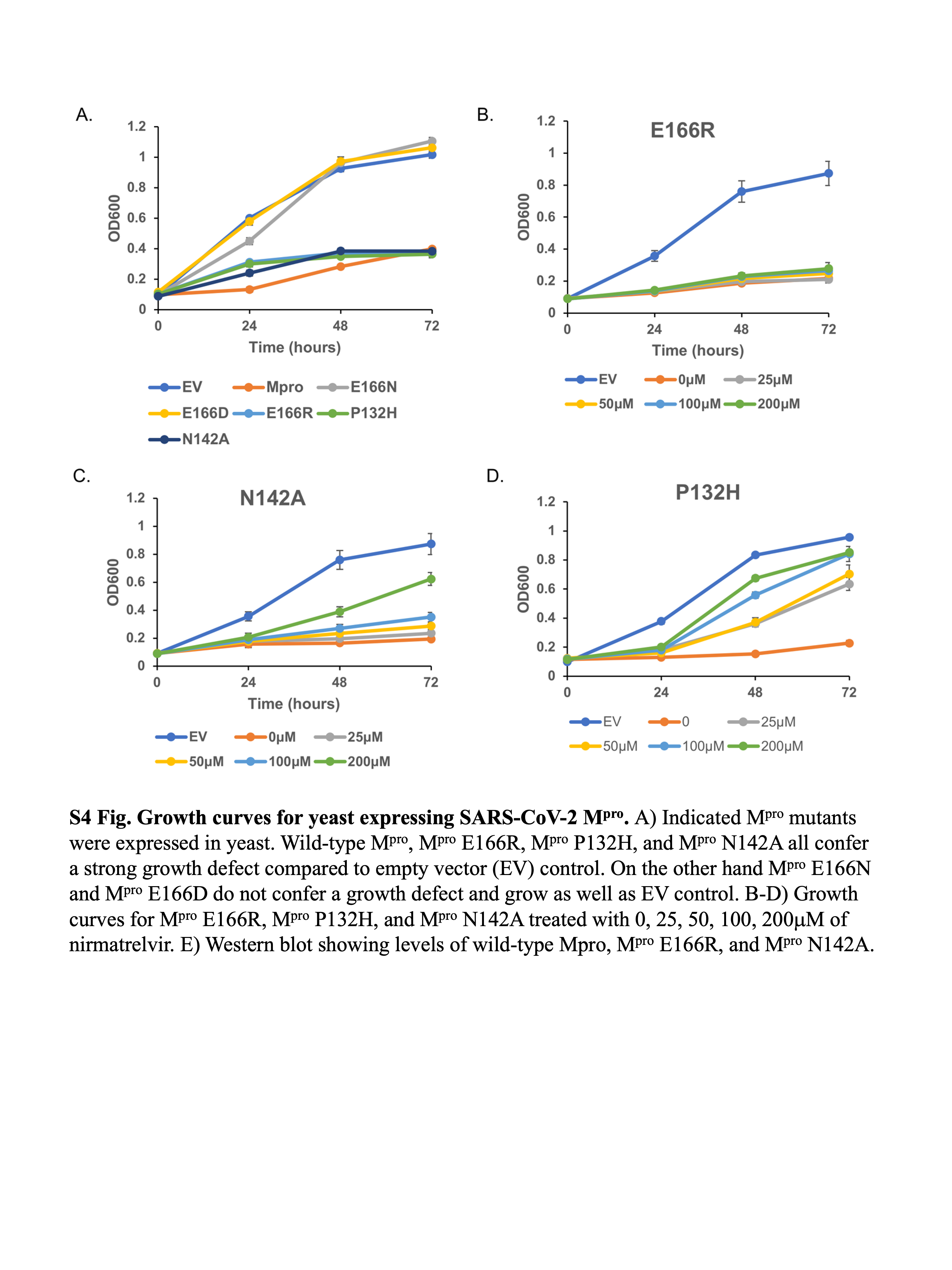

Supplement: S4 Fig — A) Indicated Mpro mutants were expressed in yeast. Wild-type Mpro, Mpro E166R, Mpro P132H, and Mpro N142A all confer a strong growth defect compared to empty vector (EV) control. On the other hand Mpro E166N and Mpro E166D do not confer a growth defect and grow as well as EV control. B-D) Growth curves for Mpro E166R, Mpro P132H, and Mpro N142A treated with 0, 25, 50, 100, 200mM of nirmatrelvir. E) Western blot showing levels of Mpro WT, E166R, and N142A. (TIFF) [file ppat.1011592.s008.tiff]

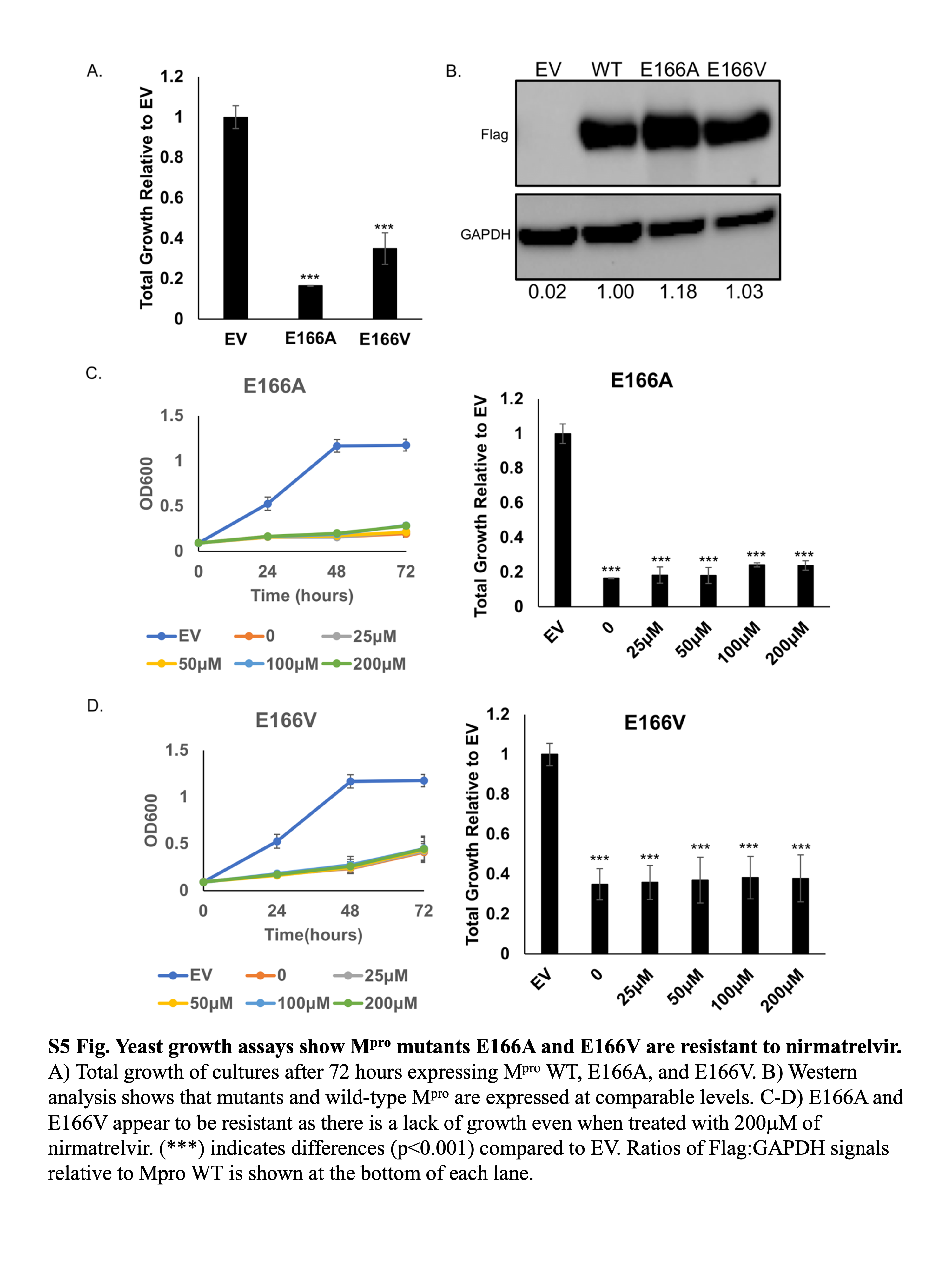

Supplement: S5 Fig — A) Total growth of cultures after 72 hours expressing Mpro WT, E166A, and E166V. B) Western analysis shows that mutants and wild-type Mpro are expressed at comparable levels. C-D) E166A and E166V appear to be resistant as there is a lack of growth even when treated with 200mM of nirmatrelvir. (***) indicates differences (p<0.001) compared to EV. Ratios of Flag:GAPDH signals relative to Mpro WT is shown at the bottom of each lane. (TIFF) [file ppat.1011592.s009.tiff]

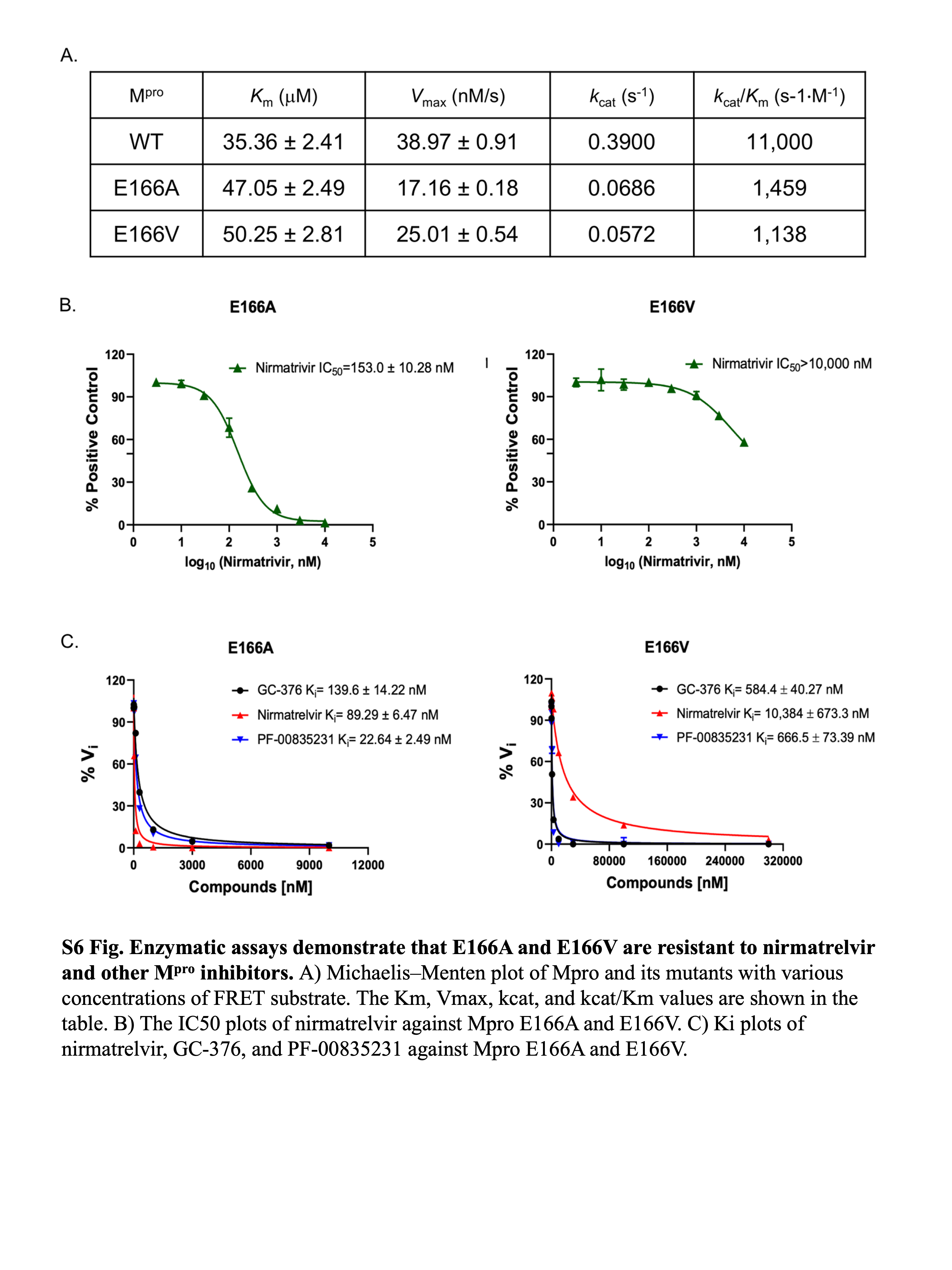

Supplement: S6 Fig — A) Michaelis–Menten plot of Mpro and its mutants with various concentrations of FRET substrate. The Km, Vmax, kcat, and kcat/Km values are shown in the table. B) The IC50 plots of nirmatrelvir against Mpro E166A and E166V. C) Ki plots of nirmatrelvir, GC-376, and PF-00835231 against Mpro E166A and E166V. (TIFF) [file ppat.1011592.s010.tiff]
